# Quality of fresh and chilled-stored raccoon dog semen and its impact on artificial insemination efficiency

**DOI:** 10.1186/s12917-016-0858-6

**Published:** 2016-10-10

**Authors:** Łukasz Jarosz, Zbigniew Grądzki, Marcin Kalinowski, Ewa Laskowska

**Affiliations:** Department of Epizootiology and Clinic of Infectious Diseases, Faculty of Veterinary Medicine, University of Life Sciences in Lublin, Głęboka 30, 20-612 Lublin, Poland

**Keywords:** Raccoon dog, Semen, Apoptosis, TUNEL assay, Mitochondrial membrane potential

## Abstract

**Background:**

The aim of the study was to evaluate the quality of fresh raccoon dog semen and raccoon dog semen stored at 4 °C. The qualitative evaluation was based on apoptosis in the sperm cells, which was tested by the Annexin V/Pi assay, the TUNEL method and JC-1. In addition, the suitability of the semen for insemination and its effect on reproduction in females were determined in relation to the time of storage.

**Results:**

During cold storage of the semen, in the samples from all groups a gradual decrease was noted in the percentage of live cells, and an increase in the percentage of cells with abnormal morphology, exhibiting changes typical of late-stage apoptosis (V^+^/PI^+^), and of necrotic cells (V^−^/PI^+^). There was a significant increase in the percentage of ApoBrDu + sperm cells, while the mitochondrial membrane potential of the sperm decreased significantly after 12 h of storage at 4 °C in the case of lower-quality semen and after 48 h in the case of semen of good quality. As the percentage of sperm with DNA and cell membrane damage increased and the mitochondrial membrane potential decreased, there was an increase in AspAT and acrosin activity. The increase in the percentage of apoptotic sperm in the raccoon dog semen stored at 4 °C resulted in a decrease in the number of females with cubs.

**Conclusions:**

Identification of apoptotic changes in sperm by flow cytometry using the annexin assay, the TUNEL assay and evaluation of mitochondrial membrane potential can be recommended for determination of the suitability of raccoon dog semen for artificial insemination. The study shows that fresh raccoon dog semen should not be used for insemination more than 48 h after collection in the case of semen of very high quality, or after more than 24 h in the case of semen of poorer quality. Cytometric methods of semen analysis should also be used to evaluate various extenders of raccoon dog semen and methods of cryopreservation in terms of ensuring sperm viability, fertilization capacity, and suitability for insemination.

## Background

The growing interest in breeding fur-bearing animals and the demand for articles of fur, including raccoon dog skins, has led to increased work on modern biotechnological methods of reproduction of this species. These measures are aimed at improving the fertility and fecundity of females through the development of new methods of semen collection and storage and the use of artificial insemination [1]. The limited success achieved in this area may be primarily due to the low quality of semen used for insemination. Raccoon dog semen has been shown to be sensitive to cold shock, which damages sperm and leads to a loss of motility and biological potential [2, 3]. These phenomena limit the possibility of frozen storage and negatively affect fertilization capacity [4, 5] Unlike frozen semen, chilled material does not require specialized treatment, and the sperm cells exhibit high motility in these conditions and ensure a high fertilization rate. A beneficial effect of chilling semen is that it reduces the metabolism of sperm cells, thereby prolonging their viability [6]. Dog semen chilled to 4–5 °C has been shown to be suitable for insemination for 48 h [7], and boar semen for 5 days [8]. Long-term chilled storage of semen, however, may lead to acrosome damage or total destruction of sperm [9]. Metabolites generated in these conditions decrease the pH of the semen, and reactive oxygen species damage the cell membrane and impair the enzymatic activity of sperm [10]. While semen does contain antioxidants, endogenous antioxidant processes do not protect the cells during long-term storage, which leads to inhibition of ATP production by sperm, damage to cellular DNA, and a decrease in or loss of motility [11].

The lack of commercially available diluents and cryoprotectants effective for raccoon dog semen often results in the deterioration of material of high genetic value. Previous research has indicated the possibility of using glycerol and EDTA as cryoprotectants in the chilling or freezing of raccoon dog semen [3]. These compounds, however, are effective only in the case of semen of high quality, in terms of motility and viability as well as microbiological contaminants [12].

Selection of raccoon dog semen for insemination has thus far been based exclusively on macro- and microscopic evaluation. In recent years apoptotic changes observed in sperm cells during cold or frozen storage of semen have been shown to reduce ejaculate quality, and may provide a new criterion for evaluating the suitability of semen for insemination [13]. Research on cattle, sheep and humans has shown that the processes of chilling or freezing and thawing semen increase the percentage of necrotic and apoptotic cells to 31–40 % [14]. The same processes in the case of boar semen raise the percentage of apoptotic cells to even 80 % in comparison with fresh semen [15]. The presence of damaged sperm in the semen affects male fertility indices and fertilization rates, as well as contributing to reproductive disorders [11, 16].

Evaluation of apoptosis in semen is particularly important in the biotechnology of animal reproduction, which entails transport of semen over long distances and thus the need to preserve it [11, 17]. In the case of raccoon dog semen, this type of research, enabling determination of changes at the molecular level in cells with seemingly normal morphology, has not been conducted. The results of such research could form the basis for improving methods of storage and preservation of raccoon dog semen or for the development of new methods.

The aim of the study was to evaluate the quality of fresh raccoon dog semen and raccoon dog semen stored at 4 °C. The qualitative evaluation was based on apoptosis in the sperm cells, which was tested by the Annexin V/Pi assay, the TUNEL method and JC-1. In addition, the suitability of the semen for insemination and its effect on reproduction in females were determined in relation to the time of storage.

## Methods

### Experimental samples

In the study we used 20 samples of semen obtained from 20 farmed raccoon dog males at the age of 2 years. The animals were in good health and normal reproductive condition and were fed according to standard recommendations for the species. The semen was obtained manually by masturbation at room temperature (25 °C) [3, 18] after the males had been accustomed to their housing and to the individual collecting the semen. The semen was collected into sterile plastic test tubes (Equimed, Poland) and stored in a water bath at 37 °C for 1 h until cooled to that temperature. The pH and volume of each ejaculate were measured. The semen samples were initially evaluated macroscopically and then examined in detail under a microscope. Sperm concentration was determined using a haemocytometer (Neubauer chamber) and a phase-contrast microscope (Nikon E200F, Equimed, Poland). Sperm motility was examined under a phase-contrast microscope (Nikon E200F, Equimed, Poland) at 400× magnification on a glass slide warmed to 37 °C [18]. The morphological tests were performed using the Diff Quik® kit (Sigma–Aldrich, Vienna, Austria), which is based on a modification of the Wright Giemsa stain and is commonly used in histological staining to rapidly stain and differentiate a variety of smears. Abnormal morphological features were determined by examining 100 spermatozoa at 400× magnification under phase-contrast microscopy. The semen samples were divided into three groups, R1 (*n* = 8), R2 (*n* = 6) and R3 (*n* = 6) (Table 1), according to sperm morphology, the percentage of undamaged cells, the percentage of cells with changes in the tail, and motility. The males in R1 had the highest percentage of undamaged sperm, the lowest percentage of sperm with changes in the tail, and the highest sperm motility. The semen of the males from group R3 was characterized by the highest percentage of sperm with tail damage and the lowest motility. In the males from group R2 a lower percentage of undamaged sperm was noted in comparison to groups R1 and R3, but motility was higher and the percentage of sperm with changes in the tail was lower than in group R3. The semen for storage was diluted to a concentration of 150 million cells per insemination dose using a modified EDTA diluent for foxes which contained 54.6 g/L anhydrous glucose, 3.75 g/L tri-sodium citrate dihydrate, 1.20 g/L sodium bicarbonate, 1.00 g/L neomycin sulfate, and 3.70 g/L of the disodium salt of ethylenediaminetetraacetic acid (EDTA), 10 % v/v glycerol [19]. The semen for insemination was stored in MINITUB straws (INSATEX-MT, Poznań).

**Table 1:** Sperm morphology of fresh raccoon dog semen, mean values and ± standard deviation (%)

Semen	N=		Motility	I	II	III	IV	V	VI	VII
R1	8	Mean (%) ±SD	79,9 10,2	69,0 7,3	5,6 3,1	10,2 5,2	3,3 2,8	5,5 2,1	0,2 0,1	1,2 0,6
R2	6	Mean (%) ±SD	61,3 8,6	32,3 4,5	1,8 1,1	22,8 6,8	15,6 6,4	23,6 3,7	1,3 0,5	1,0 0,8
R3	6	Mean (%) ±SD	30,4 11,3	41,4 7,4	0,5 0,3	33,6 5,1	16,6 7,3	12,7 2,3	0,9 0,2	0,7 0,4

Categories I-VII – sperm defects determined on the basis of microscopic examination: *I* intact sperm, *II* with a protoplasmatic droplet, *III* with a bent tail, *IV* with a coiled tail, *V* damaged sperm, *VI* with acrosomal damage, *VII* agglutinated spermatozoa. *R1*, *R2*, *R3* group of semen. *N=* number of semen samples

### Determination of AspAT and acrosin activity

AspAT and acrosin activity were determined in the plasma of the fresh and stored semen. Activity of aspartate aminotransferase was determined by the kinetic method using a kit from Alpha Diagnostics, Warsaw, and acrosin activity by the method of Kennedy et al. [20].

### Annexin V/Pi assay

An Annexin V-FITC Apoptosis Detection Kit (BD Pharmingen Poland, 556547) was used to detect the translocation of PS from the inner to the outer leaflet of the plasma membrane of fresh semen and semen stored at 4 °C. The procedure was conducted according to the manufacturer’s recommendations and as described by Anzar et al. [21].

### TUNEL assay

An APO-BRDU Kit (catalogue no. APT115; Chemicon International, Inc., Temecula, CA) was used to detect nicked DNA in fresh semen and semen stored at 4 °C as recommended by the manufacturer and as described by Anzar et al. [21].

### Assessment of mitochondrial membrane potential ΔΨm

The lipophilic cationic probe JC-1 was used to assess the mitochondrial status of the sperm. The JC-1 assay was performed as recommended by the manufacturer (Molecular Probes, Invitrogen Life Sciences, Fullerton, CA, USA) and described by Robles and Martínez-Pastor [22].

### Flow cytometric analysis

A Coulter EPICS XL flow cytometer (Coulter Corporation, Inc., Hialeah, FL) equipped with an argon-ion laser (488 nm) was used to analyse fluorescence intensities in sperm labelled with Annexin V/PI, TUNEL and JC-1. Green fluorescence (FITC) was detected with PMT2 (behind 550 DL and 525 Band Pass Filters) and red fluorescence (PI) with PMT4 (behind 600 DL and 575 Band Pass Filters). The green fluorescence due to fluorescein-labelled anti-BrdU monoclonal antibody was collected with PMT2 (behind 525 DL and 550 Band Pass Filters). The integrated and peak red fluorescence (PI) were collected through PMT3 (behind 640 DL and 610 Band Pass Filters) to measure total DNA per cell. In all assays the sperm population was identified by a combination of side-scatter (SS) and forward-scatter (FS) information. The peak fluorescence channels were determined using EPICS XL software (Coulter) and expressed on a logarithmic scale. Ten thousand cells were analysed per sample.

### Evaluation of the suitability of raccoon dog semen for insemination

Fertility of male raccoon dogs and the suitability of their semen for artificial insemination were determined by inseminating females with semen of males from groups R1, R2 and R3. The procedure was carried out using fresh semen immediately after collection and semen kept in cold storage for 12, 24 and 48 h. In total 96 females aged 3–4 were used in the study. They were selected for the experiment on the basis of a clinical examination and analysis of their reproductive history from previous breeding seasons. All inseminated females had previously given birth to live litters numbering on average 6–8 pups, and no disorders had been observed during the post-partum period, such as inflammation of the uterus, vagina or mammary gland.

Females included in the study were divided into 12 experimental groups of 8 individuals each. All of them were in oestrus, which was diagnosed by evaluation of vulval swelling to determine the optimal day for the first insemination of each female [23]. The animals were examined twice daily in February and March for signs of oestrus. Oestrus was marked by gradual swelling of the vulva and a mucopurulent discharge which was sometimes quite abundant. Artificial insemination was performed immediately after vulval swelling began to subside, the discharge had become paler and the proportion of parabasal and intermediate cells to the number of superficial and anuclear cells in vaginal smears was about one to three [24]. Artificial insemination was performed twice at intervals of 48 h by depositing sperm into the uterine lumen. The final sperm concentration in the insemination dose was 150 × 10^6^/ml, and artificial insemination was performed using a 0.5 ml MINITUBE straw (INSATEX-MT, Poznań). One hour before insemination the chilled semen was gradually heated in a water bath to room temperature.

Semen selected at random from two males of each group (R1, R2 and R3) was used for insemination. Semen from randomly selected male R1/1 was used to inseminate 4 females at each time, i.e., immediately after semen collection and after 12, 24 and 48 h of storage. Similarly, 4 females were inseminated at each time with semen from randomly selected male R1/2. The same principle was applied for the group R2 and R3 semen. In total 8 females were inseminated with semen from each group at each time (Table 2). The semen of the males was not pooled.

**Table 2:** Schema for the insemination

Group of females	Group of semen	Male	Female	Semen
1	R1	R1/1	1–4	fresh
		R1/2	5–8	
2	R1	R1/1	9–12	12 h
		R1/2	13–16	
3	R1	R1/1	17–20	24 h
		R1/2	21–24	
4	R1	R1/1	25–28	48 h
		R1/2	29–32	
5	R2	R2/1	33–36	fresh
		R2/2	37–40	
6	R2	R2/1	41–44	12 h
		R2/2	45–48	
7	R2	R2/1	49–52	24 h
		R2/2	53–56	
8	R2	R2/1	57–60	48 h
		R2/2	61–64	
9	R3	R3/1	65–68	fresh
		R3/2	69–72	
10	R3	R3/1	73–76	12 h
		R3/2	77–80	
11	R3	R3/1	81–84	24 h
		R3/2	85–88	
12	R3	R3/1	89–92	48 h
		R3/2	93–96	

After artificial insemination, females remained under clinical observation throughout the pregnancy; special attention was paid to the number of pregnant females.

### Statistical analysis

The results obtained are expressed as means ± SD. Statistical analyses were performed using Statistica 6.0 software (StatSoft, Tulsa, USA). The effect of the mean values obtained for particular parameters was estimated by Student’s t-test at *p* < 0.01 and *p* < 0.05.

## Results

### Macro- and microscopic evaluation of semen

The volume of the ejaculates obtained from the raccoon dog males ranged from 0.4 to 1.3 ml (on average 0.69 ml). The pH of the semen ranged from 6.4 to 7.6 (on average 6.93). The semen samples were white or whitish-yellow and had a watery to milky consistency. The sperm concentration in the samples ranged from 0,195 to 2,75 × 10^6^/ml (on average 0,859 × 10^6^/ml). The motility rate of the sperm in the fresh semen ranged from 30,4 % to 79,9 %, on average 57.2 %. Sperm morphology are presented in Table 1.

### Annexin/Pi assay

**Fig. 1:**
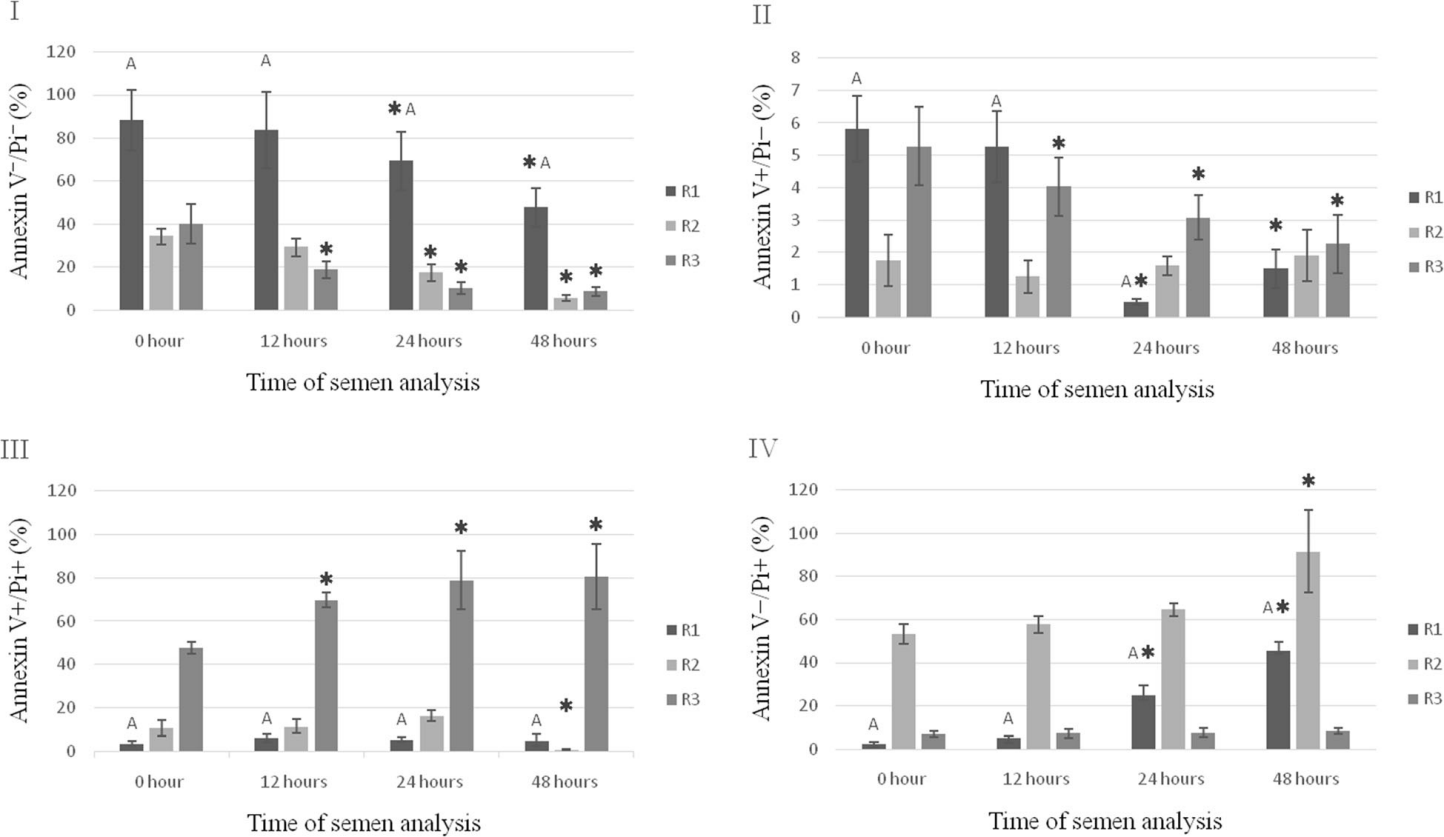
Evaluation of the semen of raccoon dogs in groups R1, R2 and R3 using Annexin V/Pi (%). **I** - percentage of living spermatozoa without apoptotic changes, **II** - percentage of spermatozoa with early apoptotic changes, **III** - percentage of spermatozoa with late apoptotic changes, **IV** - percentage of spermatozoa with necrosis. Data presented as mean and ± standard deviation. ✱ - asteriks indicate statistically significant differences at *p* < 0.05 between assay times and hour ‘0’. A - statistically significant differences at *p* < 0.01 between group R1 and groups R2 and R3. R1, R2, R3 - group of semen

The results of the assay using staining of sperm cells with annexin and propidium iodide (Annexin V/Pi) are presented in Fig. 1. The data show statistically significant differences between groups in the percentage of live sperm cells (Annexin V^−^/Pi^−^) in the fresh semen. The highest percentage of live cells was noted in group R1 (*P* < 0.01), while in groups R2 and R3 they accounted for less than 50 % of all sperm cells. During storage of the semen the percentage of live sperm cells decreased, with the greatest decrease noted 24 h after the semen was collected (*P* < 0.05). The percentage of sperm cells in late-stage apoptosis (Annexin V^+^/Pi^+^) in the fresh semen was highest in group R3, and increased in proportion to the storage time (*P* < 0.05). In the remaining groups the percentage of these cells ranged from 0.93 % to 16.32 %. The percentage of early apoptotic sperm (Annexin V^+^/Pi^−^) in the fresh semen was lowest in group R2 and did not change statistically during storage. Statistical differences were first observed after 12 h of semen storage in group R3 (*P* < 0.05) and after 24 h in group R1 (*P* < 0.05). The percentage of necrotic cells (Annexin V^−^/Pi^+^) in the fresh semen differed statistically between groups (*P* < 0.01). The highest value for this parameter was noted in group R2 (53.20 %). In group R1 the percentage of dead cells was low in the fresh semen, increasing gradually with storage time (*P* < 0.05). In group R3 the percentage of dead cells remained at the same level during semen storage, but after 48 h a statistically significant increase in this percentage was observed (*P* < 0.05).

### TUNEL

**Fig. 2:**
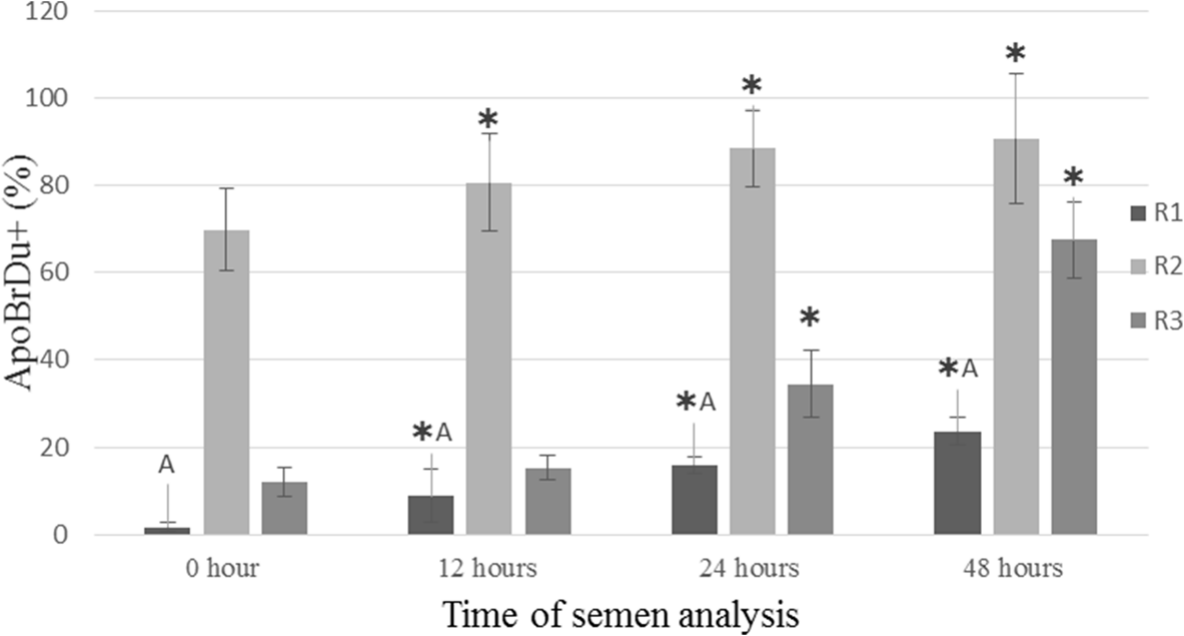
Evaluation of the semen of raccoon dogs in groups R1, R2 and R3 using FL1- PI/RNase + (ApoBrDu+). Data presented as mean and ± standard deviation. ✱ - asteriks indicate statistically significant differences at *p* < 0.05 between assay times and hour ‘0’. A - statistically significant differences at *p* < 0.01 between group R1 and groups R2 and R3. R1, R2, R3 - group of semen

The results of the TUNEL assay are presented in Fig. 2. The data show the highest percentage of DNA strand breaks in the cells of the fresh semen in group R2 (*P* < 0.05), and this percentage increased with the storage time of the sample. The lowest percentage of DNA damage was noted in group R1 (*P* < 0.01). In groups R3 and R1, 24 h after semen collection a significant increase was noted in the percentage of sperm cells with DNA damage (*P* < 0.05).

### Mitochondrial membrane potential

**Fig. 3:**
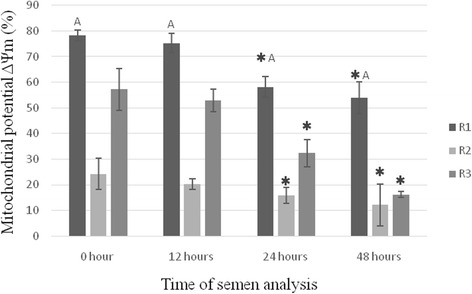
Mitochondrial potential ΔΨm (%) in semen of raccoon dogs in groups R1, R2 and R3. Data presented as mean and ± standard deviation. ✱ - asteriks indicate statistically significant differences at *p* < 0.05 between assay times and hour ‘0’. A - statistically significant differences at *p* < 0.01 between group R1 and groups R2 and R3. R1, R2, R3 - group of semen

The mitochondrial membrane potential of the sperm cells of the fresh semen was highest in group R1 (*P* < 0.01) and lowest in group R2 (Fig. 3). The differences between groups were statistically significant. In all groups the mitochondrial membrane potential of the cells of the semen decreased in proportion to the storage time, and was highest 24 h after semen collection (*P* < 0.05).

### AspAT and acrosin

**Fig. 4:**
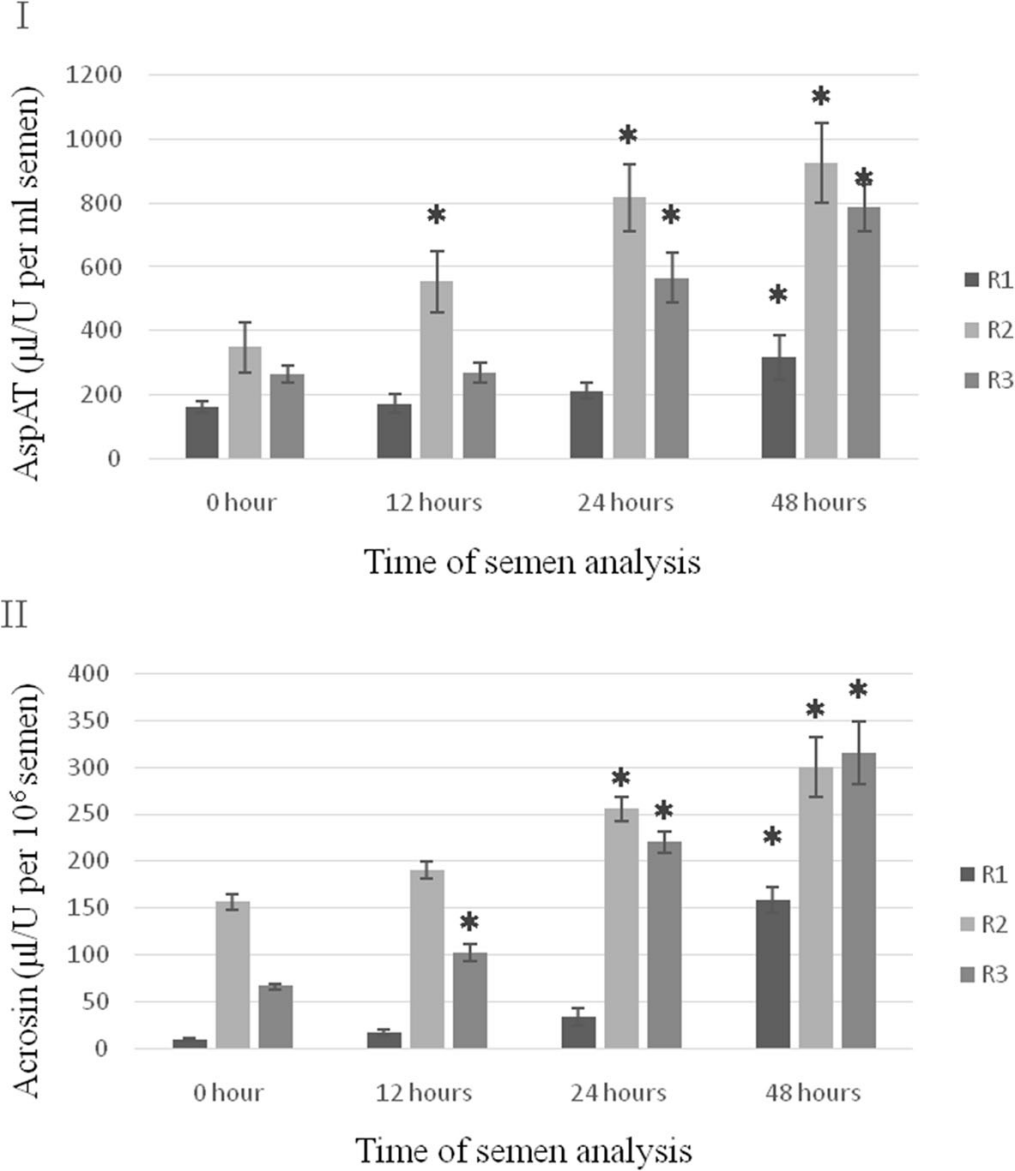
AspAT (μl/U per ml semen - **I**) and acrosin (μl/U per 10^6^ sperm - **II**) activity in raccoon dog semen in groups R1, R2 and R3. Data presented as mean and ± standard deviation. ✱ - asteriks indicate statistically significant differences at *p* < 0.05 between assay times and hour ‘0’. A - statistically significant differences at *p* < 0.01 between group R1 and groups R2 and R3. R1, R2, R3 - group of semen

AspAT activity in the plasma of the fresh semen was lowest in group R1, averaging 164.38 μl/U per ml, while the highest activity, 349.56 μl/U per ml, was noted in group R2 (Fig. 4). During storage of the semen the activity of this enzyme increased, reaching its highest value in R2, at 925.73 μl/U per ml (*P* < 0.05). A similar dependency was shown for acrosin activity. In the fresh semen it averaged 9.84 and 156.44 μl/U per 10^6^ sperm cells in groups R1 and R2, respectively. During storage of the semen acrosin activity increased in all groups, reaching its highest value 48 h after collection, i.e., 158.75–315.48 μl/U per 10^6^ cells (*P* < 0.05).

### The result of insemination

**Fig. 5:**
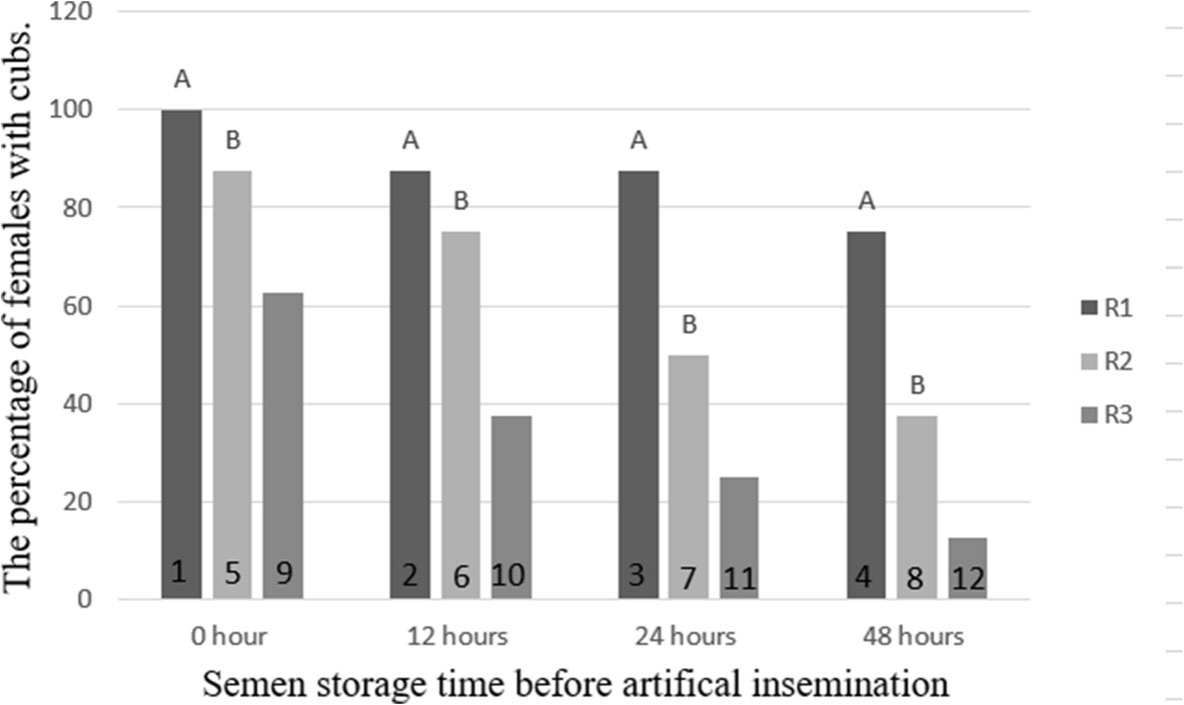
The percentage of females with cubs inseminated with fresh and chilled-stored raccoon dogs semen. The percentage of females with cubs are presented as columns. A - statistically significant differences at *p* < 0.01 between group R1 and R2, R3. B - statistically significant differences at *p* < 0.01 between group R2 and R3. R1, R2, R3 - group of semen. 1–12 - group of females

The percentage of females with cubs as a result of insemination with the semen of raccoon dogs from the three groups, fresh or stored for 12–48 h, is presented in Fig. 5. These data indicate that the percentage of females with cubs was highest in the case of artificial insemination the semen with group R1 (*P* < 0.01). The lowest percentage of pregnant females was noted following insemination with semen collected from the males of group R2. Insemination using the semen of males from group R3 was 87,5–75,0 % successful only in the case of fresh semen and semen stored up to 12 h after collection (*P* < 0.01).

## Discussion

Fertility of male and female raccoon dogs significantly influences the success of insemination, conditions the acquisition of healthy and valuable litters and affects economic outcomes in breeding. Male fertility has traditionally been diagnosed by microscopic assessment of the concentration, motility and morphology of sperm in the ejaculate. These tests provide the essential fundamental information on sperm quality [25]. Januskauskas et al., however, have shown that while many sperm characteristics determining semen quality can be evaluated in a morphological examination, it is cell membrane integrity that has the greatest influence on male fertility [26]. Therefore the results of semen analysis based on cytofluorometric evaluation of sperm viability, chromatin structure stability, and mitochondrial function can be correlated with male fertility [27–32]. Analysis of selected parameters of apoptosis, including cell membrane integrity, DNA fragmentation and mitochondrial activity in the cells of fresh semen or semen undergoing heat treatment during storage and preservation is also useful for qualitative evaluation of sperm in terms of its fertilization capacity. Cytofluorimetric analysis of raccoon dog semen also enables selection of the best-quality material for further storage and freezing and facilitates the development of effective extenders and cryoprotectants [1–3]. We used three fluorescence techniques to evaluate apoptosis and mitochondrial activity in the sperm of raccoon dogs in fresh semen and during storage at 4 °C. The results of the Annexin-V/Pi test for the fresh semen of males from group R1 were correlated with the microscopic evaluation of the semen samples. Both methods found a large percentage of live sperm with normal morphological structure and a small percentage of subpopulations of apoptotic and dead cells and cells with primary defects. During cold storage of the semen, in all groups a gradual decrease was observed in the percentage of live cells and an increase in the percentage of cells with abnormal morphology, with changes typical of late apoptosis (V^+^/Pi^+^), and of necrotic cells. None of the groups tested had a large percentage of sperm cells in early-stage apoptosis (V^+^/Pi^−^), which indicates that this stage is short and the cells quickly enter the late stage of apoptosis and necrosis. The presence of sperm cells in various stages of apoptosis and necrosis in the material shows that these phenomena play an important role in spermatogenesis and affect male fertility [2, 21]. To date no results of similar studies on raccoon dog semen have been published. However, studies using the semen of other species, including cattle, pigs and horses, confirm our observations that apoptosis of sperm cells plays a significant role in evaluation of male and female fertility, and is an important parameter of evaluation of semen used for artificial insemination [33]. The results of studies conducted on humans [34] and bulls [21] indicate that a high percentage of apoptotic cells can be treated as a marker of fertility disorders in males. In analysing the results of our study we should consider the cause of the appearance of apoptotic cells in fresh ejaculates of raccoon dogs. Apoptosis is known to be one of the mechanisms for controlling excess sperm production. In the case of testicular diseases involving dysfunction of or damage to the Sertoli cells, the process of elimination of apoptotic sperm is impaired, which results in their release into the lumen of the seminiferous tubules and an increase in their concentration in the semen. In our study the increased percentage of cells in late-stage apoptosis (V^+^/Pi^+^) in the fresh semen of group R3 is indicative of impaired elimination of such cells. A study on fresh bull semen showed that the percentage of apoptotic cells differed between individuals, suggesting that the animals differed in terms of their ability to eliminate modified cells, which influences fertility [35, 36]. This is confirmed by our results, in which fertilization rates were lower in the case of the semen of the males in groups R2 and R3, indicating low reproductive capacity in these males. Storage of semen at 4 °C does not inhibit sperm metabolism, which may lead to damage by accumulated toxic products, including free radicals. This phenomenon has also been confirmed in studies conducted on rats [37, 38]. The increased translocation of phosphatidylserine shown in the present study and the loss of mitochondrial membrane potential, particularly visible after 24 h of semen storage, indicates enhanced apoptosis affecting the suitability of the sperm cells for insemination.

A more complete picture of the phenomenon of apoptosis is obtained in laboratory studies by analysing the results of the TUNEL assay, which enables detection and evaluation of the degree of cellular DNA degradation [39, 40]. Evidence based medicine shows that sperm DNA fragmentation (SDF) tests can differentiate fertile and infertile males and that high levels of SDF are positively correlated with lower fertilization rates, impaired implantation rates and increased incidence of abortion. Evenson et al. [41] suggested that assessment of DNA integrity in sperm could be an independent marker of fertility [42]. The results obtained indicate that DNA fragmentation is an important element of assessment of the fertility of male raccoon dogs. The high percentage of ApoBrDu + sperm in group R2 indicates that the semen contained substantial numbers of cells with degraded DNA and should not be used for insemination. It is should be emphasized that the results of the TUNEL assay in this group were correlated with the results of the annexin assay, in which a high percentage of necrotic cells (V^−^/Pi^+^) was found. Thus selection of animals aimed at eliminating such males from use for reproduction can be based on the results of these two tests. Different results were obtained in the TUNEL assay for group R3, in which despite the high percentage of cells in late-stage apoptosis the percentage of cells with DNA fragmentation was small. In this group the result of insemination was also lower than in group R1, which in light of the difficulties involved in breeding raccoon dogs may be considered satisfactory. Our study also shows that chilled storage of raccoon dog semen significantly affects DNA fragmentation, as indicated by the high percentage of damaged sperm in all groups in the semen samples stored longer than 24 h. This phenomenon may be explained in part by the use of improper dilution and preservation techniques or by an inappropriate choice of extender. The results of the TUNEL assay indicate that this method of testing chilled raccoon dog semen enables reliable assessment of the fertility of males in terms of their suitability for insemination. It should be emphasized that necrotic cells becoming stained in the assay with propidium iodide (Pi) show a tendency to disintegrate, and the proteases and other cytotoxic substances released at that time may affect fertility in females by damaging the embryo or ovum. In this context the presence of a significant percentage of apoptotic cells in the fresh semen and the semen stored at 4 °C may lead to a lack of reproductive success. Analysis of the results of raccoon dog semen testing should take into account the fact that in all animal species there is a certain degree of sperm DNA damage in the ejaculate, called sperm DNA fragmentation – SDF [43–46]. A variety of processes may underlie SDF, such as oxidative stress, apoptosis or abnormalities in DNA packaging [47]. It should therefore be assumed that a small percentage of sperm cells obtained from the raccoon dogs for the present study initially had iatrogenic damage. The percentage of cells with SDF has been shown to depend on changes in temperature and on microbiological contamination of the semen [14, 48], which has been confirmed in humans infected with *Chlamydia trachomatis* and *Mycoplasma spp*. [49]. With respect to raccoon dogs, this question is still in the research phase.

Another important parameter of semen evaluation affecting male fertility is mitochondrial membrane potential, which is an indicator of the functional status of mitochondria [50]. The results indicate that the mitochondrial membrane potential of the sperm, which is an indicator of apoptosis, decreased significantly during storage of the semen at 4 °C, with the greatest impairment of mitochondrial function observed after 12 h of storage. Analysis of the percentage of ΔΨm cells following staining with JC-1 dye showed that mitochondrial function was correlated with the assessment of apoptosis using the TUNEL method and with the results of the annexin assay (Annexin-V/Pi). Detection of changes in mitochondrial membrane potential, found in the early stages of apoptosis, seems to be a good criterion for evaluation of this phenomenon in raccoon dog semen. This is confirmed by the results obtained for group R3, in which despite a low percentage of live sperm and a high percentage of apoptotic cells (V^+^/Pi^+^) in the annexin assay, their mitochondrial activity, and thus their motility, is considerable. Comparing these results with the results of the TUNEL method, we can conclude that the semen from this group of males can be used for insemination. Different observations arise from comprehensive analysis of the data obtained for group R2. The high percentage of Pi^+^ and ApoBrDu + sperm in this group and the low mitochondrial membrane potential indicate that such semen should not be used to inseminate females because it could lead to reproductive losses.

Analysis of reproductive indicators in the males from each group, shows a positive correlation between the occurrence of apoptotic changes in the semen and insemination rate. The results also suggest that we cannot rule out the possibility of fertilization of the ovum by sperm with lower biological value, such as apoptotic cells, which are often responsible for embryonic death in the early stage of development. Such a relationship was found in the present study in the case of the semen of the R3 group, whose use for insemination 12 h after collection resulted in a fertilization rate of over 50 %.

The fertilization capacity of sperm largely depends on acrosome enzymes [3, 18, 51]. Evaluation of the relationship between sperm motility, expressed as mitochondrial membrane potential, and acrosin activity in the semen plasma showed that the activity of the enzyme increased as the motility of the raccoon dog sperm decreased. Similar observations were reported by Froman et al. [52], who analysed sperm motility in the silver fox and found a close correlation between acrosin activity and the degree of sperm damage. In our study an increased concentration of this enzyme in the raccoon dog semen plasma was correlated with an increase in the percentage of sperm with DNA and cell membrane damage and with a decrease in mitochondrial membrane potential. A similar relationship was found in the case of aspartate aminotransferase concentration, which was lower in the plasma of the fresh semen than in the stored semen, and varied between groups. These results may indicate that raccoon dog semen is not well suited for storage, and AspAT concentration may be treated as a marker of the degree of damage to the enzymatic apparatus of the sperm, reflecting its biological value. The study shows that fresh raccoon dog semen should not be used for insemination more than 48 h after collection in the case of semen of very high quality, or after 24 h in the case of semen of inferior quality.

## Conclusions

To sum up, identification of apoptotic changes in sperm by flow cytometry are useful in evaluating the quality of raccoon dog semen. The annexin and TUNEL assays and evaluation of the mitochondrial membrane potential of raccoon dog semen can be recommended for determination of the suitability of raccoon dog semen for insemination and for evaluation of the fertility of males used to rebuild the foundation stock. Cytometric methods of semen analysis should also be used to evaluate different extenders of raccoon dog semen and cryopreservation methods in terms of ensuring viability of sperm, fertilization capacity, and suitability for insemination.

## Abbreviations

Apo: Apoptosis
AspAT: Aspartate aminotransferase
BrdU: 5-bromo-2′-deoxyuridine
EDTA: Ethylenediamine tetraacetic acid
FITC: Fluorescein isothiocyanate
JC-1: 5,50,6,60-tetrachloro-1,10,3,30-tetraethylbenzimidazolyl-carbocyanine iodide
Pi: Propidium iodide
TUNEL: Terminal Deoxynucleotidyl Transferase-mediated d-UTP Nick End Labeling
ΔΨm: Mitochondrial membrane potential
